# Meropenem–Vaborbactam for the Treatment of Post-Neurosurgical Meningitis Caused by KPC Producer Klebsiella Pneumoniae: A Case Report and Review of the Literature

**DOI:** 10.3390/antibiotics13040331

**Published:** 2024-04-05

**Authors:** Leonardo Francesco Rezzonico, Francesco Peracchi, Marta Vecchi, Gabriele Bassi, Marco Merli, Nicholas Brian Bana, Giovanna Travi, Fulvio Crippa, Massimo Puoti

**Affiliations:** 1 School of Medicine, University of Pavia, 27100 Pavia, Italy; leonardofrance.rezzonico01@universitadipavia.it; 2 Department of Infectious Disease, ASST Grande Ospedale Metropolitano Niguarda, 20162 Milan, Italy; marta.vecchi@ospedaleniguarda.it (M.V.); marco.merli@ospedaleniguarda.it (M.M.); n.bana@campus.unimib.it (N.B.B.); giovanna.travi@ospedaleniguarda.it (G.T.); fulvio.crippa@ospedaleniguarda.it (F.C.); massimo.puoti@ospedaleniguarda.it (M.P.); 3 School of Medicine and Surgery, University of Milano-Bicocca, 20126 Milan, Italy; 4 Intensive Care Unit, ASST Grande Ospedale Metropolitano Niguarda, 20162 Milan, Italy; gabriele.bassi@ospedaleniguarda.it

**Keywords:** KPC, carbapenem-resistant *Enterobacterales*, meropenem–vaborbactam, meningitis, central nervous system infection

## Abstract

Meningitis and ventriculitis, due to carbapenem-resistant *Enterobacterales*, are frequently associated with significant morbidity and mortality. In the case of multi-drug-resistant pathogens, it is necessary to consider the limited susceptibility profile as well as the penetration of the antimicrobials into the brain. Limited data are available regarding the treatment of central nervous system infections caused by carbapenem-resistant *Enterobacterales*. We report a study of a patient treated with meropenem–vaborbactam in the case of post-neurosurgical meningitis due to carbapenemase-producing *Klebsiella pneumoniae* (*CPKP*).

## 1. Introduction

Post-neurosurgical infections are severe complications leading to high mortality and neurological dysfunction. The infection rate ranges from 4.6 to 25%. In addition, the shunting and drainage of cerebrospinal fluid (CSF) and intrathecal infusion pumps may increase the risk of infections [1]. Both Gram-negative and Gram-positive bacteria may be associated with central nervous system (CNS) infections *Enterobacterales*, where non-fermenting Gram-negative bacilli and *Staphylococcus* spp. are the most frequently isolated [2]. Unfortunately, the increased use of broad-spectrum antibiotics in clinical practice has led to an increase in intracranial infections caused by resistant Gram-negative bacteria, with a particular concern, especially regarding multi-drug-resistant (MDR) and extensive drug-resistant (XDR) germs [3]. In the setting of CNS infections, carbapenem-resistant *Enterobacterales* (CRE) are the most difficult to treat, not only due to the site of infection, as the low permeability of blood–brain barrier (BBB) limits the penetration of various antimicrobials into the CSF [4], but also due to the lack of highly efficacious therapeutic options.

New combinations of cephalosporins or carbapenem with beta-lactamase inhibitors may be valid options to treat MDR pathogens, such as meropenem/vaborbactam (MEV) or ceftazidime/avibactam (CZA) [5,6]. In particular, MEV may be a good choice for CNS infection when therapeutic options are limited. Few reports on the therapeutic role of MEV in CNS infections are available, even if data on microbiological eradication are not always reported [7,8]. Among these new molecules, intrathecal (ITH) or intraventricular (IVT) antibiotic administration remains a valuable option in association with intravenous therapy, especially if the MDR/XDR pathogen is suspected or isolated [9].

We report here the case of healthcare-associated meningitis due to a carbapenemase-producing Klebsiella pneumoniae (CPKP) successfully treated with MEV and also present a review of the literature. 

## 2. Case Report

Our patient was a 59-year-old male living with HIV, on an antiretroviral regimen consisting of dolutegravir + lamivudine, with a CD4+ count of 270 cell/microL (ref. value 493–1666 cell/microL) (CD4/CD8 ratio: 0.79, ref value 1.2–2.6) and persistent negative viral-load at the time of observation. He previously experienced a long course of HCV infection with no signs of cirrhosis, successfully treated with antivirals, reaching sustained virological response. The patient was also affected by chronic heart failure (New York Heart Association-NYHA class 2–3) and on dual antiplatelet therapy due to recent coronary stent placement after myocardial infarction, non-insulin-dependent diabetes mellitus, previous sigmoidectomy in diverticular disease complicated by entero-urinary fistula, severe cerebral vasculopathy and a previously drained epidural hematoma that resulted in tetra-paresis. The patient was referred to our center from a secondary hospital because of a peri mesencephalic sub-arachnoid hemorrhage due to the disruption of a temporal artery aneurism with subsequent hydrocephalus.

An external ventricular drainage (EVD) was initially placed to manage liquoral hypertension. The patient then underwent a subsequent neurosurgical treatment (clip placement on the vertebral aneurysm, which was the cause of the bleeding). The procedure was well tolerated and without postoperative complications, besides a significant increase in myocardial enzymes and pro-B type natriuretic peptide without consistent acute heart failure or hemodynamic instability. The patient was, therefore, transferred from the neurological intensive care to the neurosurgical ordinary ward due to his stable neurological condition; he was apyretic, alert, and showed a response to simple stimuli with spontaneous breathing. The EVD was removed on day 20, with subsequent clinical and radiologic improvement documented by a brain CT scan. The day after the shunt removal, the patient experienced a rapid clinical deterioration with signs of meningism and the onset of a comatose state with the necessity of orotracheal intubation. The patient was re-admitted to the intensive care unit, and a diagnostic lumbar puncture was performed, documenting a CPKP infection with altered liquor parameter (white blood cells 6002/mm^3^, glucose < 2 mg/dL, protein > 600 mg/dL and lactate 18.4 mmol/L). CPKP was initially detected during the film-array test and subsequently confirmed at standard culture. The patient was started on intravenous (IV) MEV (2 + 2 g every 8 h infused over 3 h). IV gentamicin (320 mg daily) was added as part of combination therapy for MDR bacteria, waiting for blood culture results. To note, the patient had no known rectal or respiratory colonization by MDR pathogens at screening performed during their entire in-hospital stay. Blood cultures were negative. The patient became apyretic after 72 h of antimicrobial treatment. The therapeutic drug monitoring (TDM) of gentamicin and meropenem was performed to assess proper serum levels (2 mcg/mL at trough sample for gentamicin and 4.4 mcg/mL at peak sample for meropenem). After 5 days of antibacterial systemic-combined therapy, an improvement in CSF lactate was documented, with other inflammatory markers broadly stable. Serum inflammatory markers improved: the procalcitonin level lowered from 1.78 to 0.39 ng/mL, and total leukocytes dropped from 16.2 to 8.81 × 10^9/L. Despite efforts, the patient’s neurological state was still compromised, with only slight improvements after lowering drug-induced deep sedation. According to these clinical and biochemical data, on the 5th day of antibiotic treatment, a new EVD was placed to start intrathecal gentamicin (8 mg daily), while systemic gentamicin administration was interrupted. At the time of the EVD position, cerebrospinal fluid was still cloudy, and opening pressure was high (18 cmH_2_O). Both a microscopy examination and CSF culture-confirmed CPKP presence. On day 2 of intrathecal therapy, gentamicin TDM was assessed from the CSF sample to confirm adequate therapeutic levels (2.8 mcg/mL, trough sample). The patient was subsequently kept on IV meropenem–vaborbactam and intrathecal gentamicin with gradual neurological improvement, reaching complete respiratory weaning and resolution of aphasia though a residual more pronounced tetra-paresis, compared to the baseline, was observed. Intrathecal gentamicin was stopped at day 10 once the CSF culture was confirmed negative with a substantial normalization of CSF parameters (white blood cells 29/mm^3^, glucose 44 mg/dL, protein 30.2 mg/dL and lactate 1.9 mmol/L). See Table 1 for all CSF sample biochemical and microbiological data. EVD was consequently removed to avoid further additional infective risk. A full cycle of 21 days of MEV was completed. The patient was then admitted to a neurorehabilitation center. Up until now, four months after the CNS infection, no relapse has occurred.

## 3. Case Discussion

Carbapenemases are carbapenem-hydrolyzing β-lactamases conferring resistance to a broad spectrum of β-lactam substrates, including carbapenems. Carbapenemases are included in five major families as follows: KPC (Klebsiella pneumoniae carbapenemase; Amber class A), VIM (Verona integron-encoded metallo β-lactamases), IMP (imipenemase), NDM (New Delhi metallo- β -lactamases, Ambler class B) and OXA-48-group carbapenemases (Ambler class D) [10]. Only a few antibiotics are currently active against carbapenem-resistant pathogens. Colistin is one of the active drugs, but its toxicity, pharmacokinetic characteristics, and risk of resistance when used in monotherapy limit its use. Thus, CRE infections are a challenging condition because few treatment options are available. While new drugs such as CZA, MEV, imipenem/cilastatin/relebactam, and cefiderocol are valuable treatments against KPC producers’ pathogens, in the setting of CNS infections, the blood–brain barrier may reduce antimicrobial CSF concentrations, negatively affecting their ability to control ongoing infection. IDSA guidelines express a slight preference for the use of MEV over CZA for the treatment of KPC-producing organisms, but both are listed as options for this indication, and no specific indication for SNC infection is indicated [11]. The use of MEV against CZA may be supported by the strong affinity of vaborbactam for KPC, with a higher barrier for developing resistance. In the initial randomized phase III open-label trial [12] of MEV use for serious infections, no MEV resistance was observed, which differs from CZA. Athans et al. [13] described the successful treatment of CPKP bacteriemia and liver abscess in a liver transplant recipient after CZA treatment failure due to the emergence of resistance. 

Healthcare-associated infections are frequently associated with MDR pathogens. The rate of infective complications related to cranial surgeries was reported at 0.8% in a large series [14]. A higher infective rate was reported in patients carrying an EVD; in this setting, a systematic review showed a range of positive CSF cultures ranging from 2.3 to 23% [15]. Furthermore, EVD-related meningitis may occur even after shunt removal, as highlighted in a study where 23% of EVD-associated infections happened between 1 and 10 days after device removal [16]. While Gram-positive organisms are usually more highly represented (mainly *S. aureus*, coagulase-negative *staphylococci*, *Cutibacterium acnes*, etc.), the number of infections sustained by Gram-negative bacteria is still relevant and carries a risk of antimicrobial resistance due to involved pathogens that are prone to become non-susceptible to antibiotics under antimicrobial pressure, such as *Enterobacterales*, *P. aeruginosa* and the *A. baumannii complex* [17]. 

MEV is a combination of carbapenem with a non-beta-inhibitor of class A and C serine beta-lactamases, such as KPC [18]. No pharmacokinetic studies have been performed to assess vaborbactam penetration into CNS. Therapeutic drug monitoring (TDM) studies on meropenem penetration in post-neurosurgical meningitis confirmed an acceptable CNS concentration. The optimal meropenem CSF penetration is related to a high plasma concentration (2.4 ± 0.3 mg/L, 17.6% ± 7.3% CSF/plasma concentration) when 2 g every 8 h is administered. Continuous infusion has been reported beneficial in attaining appropriate LCR levels of meropenem [19]. A recent literature review comparing the CSF/serum ratio documented the higher penetration of meropenem than that of ceftazidime (2.7–15% and 10.7–21%, respectively); meanwhile, a substantial lack of data on the new beta-lactam–beta-lactamase (BLBLI) inhibitor association remains [20].

There are limited data on CZA use in difficult-to-treat CNS infections due to KPC K. pneumoniae reported concerning poor CNS penetration and PK/PD optimization [21].

Beta-lactamase inhibitors demonstrated a wide range of penetration into CNS, with an elevated risk of reaching CSF suboptimal concentrations and subsequent treatment failure [20,22]. Healthy volunteer (HV) CZA CNS penetration was reported in a range between 0.31–0.32 and 0.32–0.35 related to plasma concentration. No data on HV or MEV are reported [23].

A report on avibactam PK/PD in post-neurosurgical infection showed that the standard dosage of CZA (2.5 mg of q8h in continuous infusion) determined a CSF avibactam concentration below 4 mg/L and TDM was required to obtain the PK/PD target, increasing the CZA dose up to 5 g of q8h in continuous infusion [22]. Another concern related to BLBLI, and particularly CZA, is the reduction in BBB inflammation that characterizes the first phase of CNS infection, which may decrease antibiotics penetration [20].

Vaborbactam possesses a similar molecular weight compared to avibactam (297 vs. 265 g/mol) while displaying higher protein binding (33 vs. 8.2%), resembling tazobactam characteristics more closely. A higher binding protein is generally related to a reduced capability to surpass BBB [19]; previous studies showed that piperacillin–tazobactam was effective only in the treatment of CNS infections sustained by pathogens with a minimum inhibitory concentration (MIC) less than or equal to 0.5 microg/mL [24]. Vaborbactam CSF penetration data are needed.

Considering the susceptibility pattern of our CRKP, we opted to use MEV because of its more favorable MIC (CZA MIC 8/4 microg/L vs. MEV MIC ≤ 0.06 microg/L), increasing the probability of achieving a more effective pharmacodynamic target (ideally an MEV CSF concentration at least 4-fold above the pathogen MIC) compared to CZA. To the best of our knowledge, this is one of the first studies to document this in this field.

The relationship between systemic combination therapy and better outcomes is still not clear [25], although, in the case of complicated infections or MDR pathogens, combination therapy has to be considered [26]. Fosfomycin may represent a good partner for MDR infections, even when CNS is involved. Fosfomycin is a small polar peptide with a very simple structure and a low molecular weight (138 g/mol), favoring good penetration in various tissues, including CNS [27]. Moreover, it showed synergism with beta-lactams [28]. Unfortunately, CRKP isolated in our case test showed an agar dilution with unfavorable MIC to fosfomycin (MIC > 256 mg/L). Consequently, due to the severity of the infection and the limited therapeutical options, intrathecal gentamicin was added to provide greater bactericidal activity. Even if intrathecal gentamicin is related to a higher risk of CNS adverse events [22], its addition to meropenem allows a greater post-antibiotic effect that, in severe infection with poor prognosis, may be beneficial [20]. American guidelines recommend considering an ITH/IVT antibiotic injection when IV therapy administration is not effective after 48–72 h and intracranial infection is very severe [9]. 

The severity of our case of infection was underlined by CSF samples with the evidence of hypoglycorrhachia (<2 mg/dL) and hyper-proteinorrhachia (>600 mg/dL) both on the day of diagnosis and after five days of treatment. Hypoglycorrhachia (<50 mg/dL) was found to be a negative prognostic factor in Gram-negative post-neurosurgical meningitis. The prognosis was even worse when this parameter was confirmed after 48 h of treatment [29]. In our patient, after five days, the CSF–glucose level was still less than 2 mg/dL. CSF. The microscopy became negative after seven days of therapy, but the first negative culture was obtained only on the 14th day of treatment. Intrathecal gentamicin was discontinued when a negative CSF culture was confirmed. Then, the treatment was completed with MEV alone. Unfortunately, we were unable to assess MEV levels to evaluate his CNS penetration. The combination therapy used does not allow us to fully assess the impact of each single drug. However, our CSF samples showed the microbiological eradication of CRKP, suggesting the efficacy of our approach. 

Recent meta-analyses [30] conducted on post-neurosurgical infection showed how intravenous therapy combined with an ITH/IVT antimicrobial administration promotes clearance and reduces mortality in the MDR/XDR subgroup. Notably, the CRE infection 30-day mortality rate was significantly lower (8.7% vs. 33.3%) when ITH/IVT effective therapy was associated with systemic once [31]. In these studies, an antibiotic sensibility test before starting the ITH/IVT administration appeared of particular importance [32]. Moreover, ITH/IVT administration led to improvement in clinical symptoms (incidence of fever, neck stiffness, and GCS score) and cerebrospinal fluid biochemical indicators (increment of glycorrhachia and reduction in proteinorrhachia). Interestingly, no differences in mortality or in hospital stay were observed in Gram-negative multi-sensible bacterial infection comparing combination therapy to IV therapy alone. The days of CSF sterilization and hospital stay were significantly longer using the combined therapy compared to IV therapy, which correlates to more severe infections in patients exposed to combination therapy [30].

The decision to monitor trough gentamicin during ITH administration was based on data suggested that even trough aminoglycoside TDM allowed CSF/pathogen MIC to be assessed; furthermore, these data suggest that performing TDM immediately before the next intraventricular dose allowed a reduction in manipulating the EVD, with an associated reduction in further infection episodes [33].

Particularly, aminoglycosides are characterized by an elevated bactericidal effect and synergistic effect with beta-lactams [20,34]. Due to a sensibility test confirming our CPKP, ITH gentamycin administration appears in our case as a good option combined with MV. MEV was the only drug used for the full 21-day course as per the guidelines [35]. Evaluating the clinical and biochemical resolution, the absence of relapse and aminoglycosides are generally considered a savage option and is reasonable to believe that MEV played a major role in the clinical cure of the patient.

## 4. Conclusions

More data are necessary to guide the choice of treatment of CNS infections caused by multi-drug-resistant pathogens, and future pharmacokinetic and pharmacodynamic studies focusing on CNS/CSF penetration would be helpful. It is reasonable to consider MEV as a potential agent to treat CNS infections sustained by CRE, which is a particular setting in which treatment options are scarce. This choice may be motivated by meropenem SNC’s greater penetration compared to other beta-lactam molecules and MEV’s activity against KPC-producing organisms. This scenario underlines the increasing necessity of antimicrobial TDM, especially in difficult clinical contexts. The addition of intrathecal therapy with aminoglycosides or colistin might be considered in the setting of MDR pathogens due to the unfavorable PK/PD of systemic therapy and for the synergistic effect this treatment might provide.

## Figures and Tables

**Table 1:** Cerebrospinal fluid sample features.

CFS Sample	Microscopy	Culture	WBC (cell/mm^3^)	Glucose (mg/dL)	Protein (mg/dL)	Lactate (mmol/L)
Day 0	Gram negative	CRKP	6002	<2	>600	18.4
Day 5	Gram negative	CRKP	28744	<2	>600	12.5
Day 7	Negative	CRKP	n.a.	n.a.	n.a.	n.a.
Day 14	Negative	Negative	29	44	30.2	1.9

Abbreviations: CSF: cerebrospinal fluid; WBC: white blood cells; CRKP: carbapenemase-producing Klebsiella pneumoniae; n.a.: no available data.

## Data Availability

Data are available from the corresponding author upon reasonable request.
